# The Role of Latissimus Dorsi Myocutaneous Flaps in Secondary Breast Reconstruction After Breast-Conserving Surgery

**Published:** 2013-06-11

**Authors:** Koichi Tomita, Kenji Yano, Akimitsu Nishibayashi, Megumi Fukai, Miwako Miyasaka, Ko Hosokawa

**Affiliations:** Department of Plastic and Reconstructive Surgery, Graduate School of Medicine, Osaka University, Osaka, Japan

## Abstract

**Objective:** Secondary reconstruction after breast-conserving surgery is generally challenging because of the nature of irradiated tissue. The aim of this study was to validate the use of latissimus dorsi myocutaneous (LDM) flaps for secondary breast reconstruction after breast-conserving surgery. **Methods:** Fifteen consecutive patients who underwent secondary reconstruction with an LDM flap after breast-conserving surgery were included in the study. The esthetic outcome in comparison with the contralateral breast was evaluated by observer assessments consisting of 7 criteria. In addition to comparing pre- and postoperative scores for each criterion, factors affecting overall esthetic outcome were analyzed. **Results:** There was no major recipient- or donor-related complication. In 13 patients, the skin paddle of the LDM flap was exposed to the skin surface. In all patients, overall esthetic scores increased postoperatively. Age, period between breast-conserving surgery and LDM flap, body mass index, or preoperative breast size did not affect the overall esthetic outcome. Tumors in the lower quadrants tended to result in poorer esthetic scores, especially in breast shape and scar (*P* = .04 and .02, respectively). **Conclusions:** Given their high vascularity and moderate flap volume, LDM flaps could be a reliable option for secondary breast reconstruction after breast-conserving surgery. Although exposure of skin paddle to the skin surface is inevitable in most cases, esthetic improvement could be achieved, including the breast scar. On the contrary, immediate reconstruction would certainly be more desirable, especially in cases of tumors in the lower quadrants.

Breast-conserving surgery (BCS) has become a well-accepted procedure for most women with breast cancer. In addition to high survival rates comparable to radical mastectomy, a large portion of the breast can be preserved in BCS.^1^ However, around 30% of BCS reportedly results in unsatisfactory late esthetic results, especially in patients who had more than 20% of their breast volume resected.^2^ Since BCS is generally performed in combination with postoperative radiation therapy, surgical reconstruction after BCS is usually difficult because of postirradiation fibrosis, and the esthetic results are often unpredictable.^3^

Given the nature of irradiated tissue such as delayed healing, mammoplasty, using local tissue alone, likely results in a high complication rate, and therefore, the use of a well-vascularized flap is desirable. The latissimus dorsi myocutaneous (LDM) flap was first described by Tansini^4^ in 1896, and it has become a well-established procedure for breast reconstruction.^5^^,^^6^ Because of its advantages such as reliable vascularity, low complication rate, and technical ease, the LDM flap has become the most frequently used procedure in immediate breast reconstruction after BCS in our institution.^7^^-^9 In this study, to investigate whether the LDM flap could also be a good option for secondary breast reconstruction after BCS, we reviewed 21 consecutive patients in whom unilateral breast correction after BCS was performed with the LDM flap. In 15 patients, the esthetic outcome in comparison with the contralateral breast was evaluated by means of observer assessment of 7 criteria contributing to the overall outcome, and comparison between pre- and postoperative scores for each criterion was performed. Furthermore, factors affecting the overall esthetic outcome were analyzed.

## METHODS

### Patient selection and surgical procedure

We reviewed consecutive 21 patients having undergone unilateral BCS and postoperative radiation therapy, followed by secondary breast reconstruction with the LDM flap at Osaka University Hospital between April 2001 and February 2012. Inclusion criteria for the secondary reconstruction with the LDM flap were patients with mammary defect more than approximately 20% and significant asymmetry of the nipple-areola complex (NAC) position. Exclusion criteria were patients whose thoracodorsal vessels were damaged in the initial BCS. Among those patients, 15 patients whose preoperative and at least 1-year follow-up photographs were available were included in the study. The patients’ mean age was 51.3 years (range: 39-64 years), and the mean period between BCS and LDM flap reconstruction was 42.3 months (range: 7-102 months). The mean follow-up period was 56 months (range: 12-141 months). Profiles of the 15 patients are summarized in Table 1. In reconstructive surgery, the LDM flap was elevated in the lateral position, followed by transferring the flap to the affected breast in the supine position. In 13 patients, the skin paddle of the LDM flap was exposed to the skin surface because of an extensive contracture of the original skin envelope. In all cases, NAC was preserved in BCS, and no secondary procedures were performed before taking a standardized photograph as described hereafter, except for one patient (no. 7). In this patient, the skin paddle was resected secondarily after reconstruction with the LDM flap.

### Photographic assessment

Before and after reconstructive surgery (at more than 1 year after breast reconstruction), standardized photographs were taken with the patients standing upright and with their hands on each iliac crest for an objective photographic assessment. Frontal and oblique (30° left and right) photographs were independently evaluated by 3 blind assessors (nonoperative plastic surgeons). Seven criteria were assessed on a 3- or 2-point scale,^8^ which was approved by the Japanese Breast Cancer Society. Criteria included the symmetry of breast size, breast shape, appearance of the breast scar, NAC size and shape, NAC color, NAC position, and the most inferior point of the breast (Table 2). For each criterion, average scores of the 3 assessors were used. Finally, a linear *visual analog scale* from 0 to 10 was calculated as a sum of the scores of the 7 criteria. For statistical analysis, scores of 9 to 10 were considered “excellent,” 7 to 8 “very good,” 5 to 6 “good,” 3 to 4 “fair,” and 0 to 2 “poor.” Factors associated with the patient's profile (age, period between BCS and LDM flap, body mass index, preoperative brassiere cup size, and vertical tumor location) were assessed in relation to pre- and postoperative esthetic outcome. For each factor, data that were missing or considered inappropriate (such as tumor location at the center) were excluded from the analysis.

Statistical analysis was performed by using Statcel version 3 (OMS, Saitama, Japan). Data were analyzed by using the Mann-Whitney *U* test or Spearman rank correlation coefficient, where indicated. *P* < .05 was considered significant.

## RESULTS

Overall, there was no major donor-site and recipient-site complications, including flap necrosis, infection, wound rupture, or hematoma formation. The patients felt no limitation in normal life and no complaint to the donor-site scar after flap harvest. The most frequent minor complication was donor-site seroma in 4 patients, which was treated with conservative therapy.

Photographic assessment revealed that among 15 patients, 1 (6.7%) was ranked as very good, 1 (6.7%) as good, 3 (20.0%) as fair, and 10 (66.7%) as poor before reconstructive surgery. After reconstruction, 4 (26.7%) were ranked as excellent, 4 (26.7%) as very good, 5 (33.3%) as good, and 2 (13.3%) as fair. No patient was ranked as poor after reconstruction. There was a significant change in the overall esthetic assessment before and after reconstruction (*P* < .0001; Mann-Whitney *U* test). The score for each criterion also significantly increased after reconstruction (*P* < .001, for each) except for NAC size, shape, and color (Table 3).

**Figure 1:**
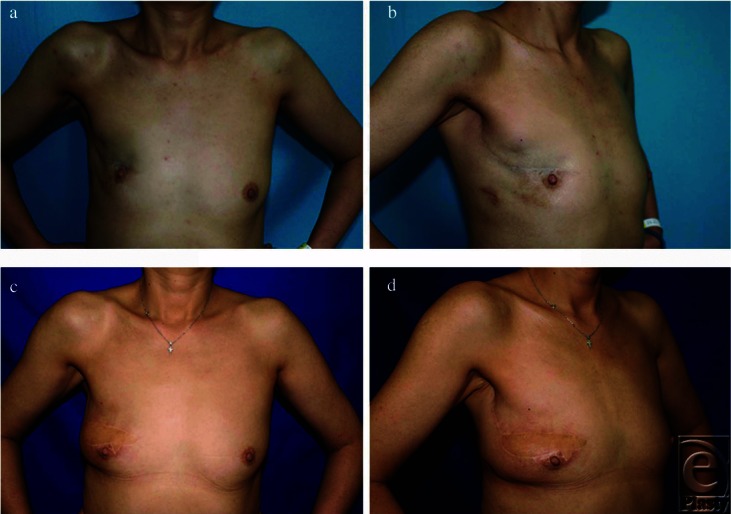
Preoperative frontal (*a*) and right oblique (*b*) views of patient no. 5 (preoperative brassiere cup size, A) after right upper quadrant breast-conserving surgery. Postoperative photographs 20 months after secondary reconstruction with a latissimus dorsi myocutaneous flap (*c* and *d*). Overall esthetic assessment improved from “poor” to “very good.”

**Figure 2:**
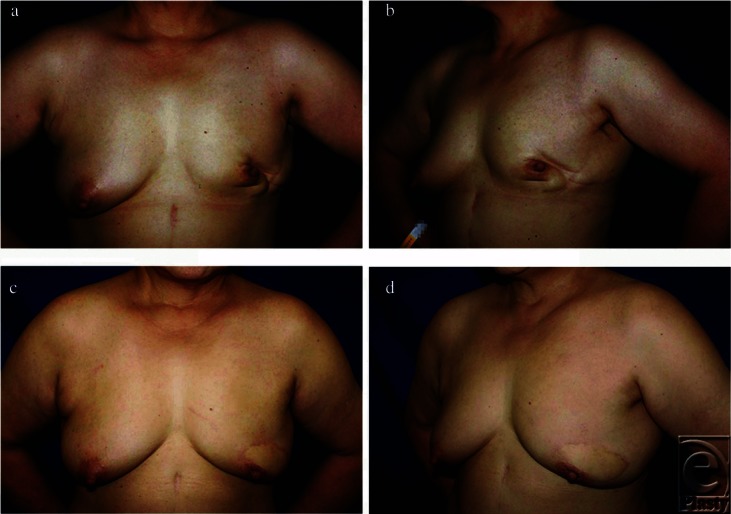
Preoperative frontal (*a*) and left oblique (*b*) views of patient no. 3 (preoperative brassiere cup size, C) after left upper quadrant breast-conserving surgery. Postoperative photographs 96 months after secondary reconstruction with a latissimus dorsi myocutaneous flap (*c* and *d*). Overall esthetic assessment improved from “poor” to “excellent.”

**Figure 3:**
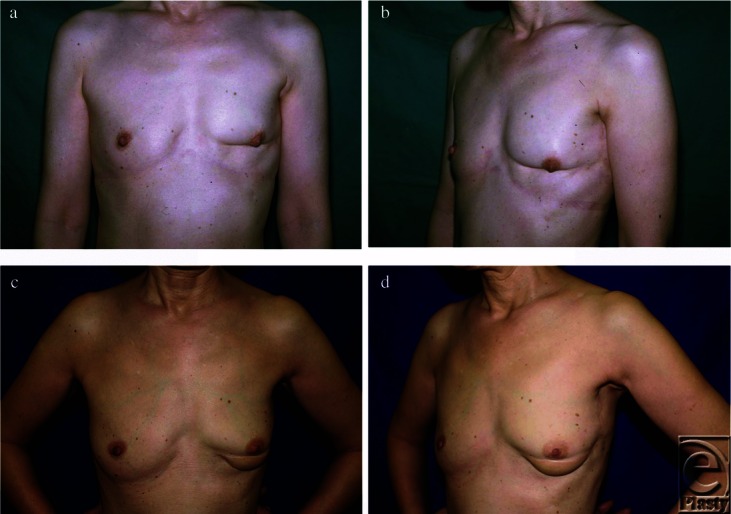
Preoperative frontal (*a*) and left oblique (*b*) views of patient no. 14 (preoperative brassiere cup size, A) after lower quadrants breast-conserving surgery. Postoperative photographs 48 months after secondary reconstruction with a latissimus dorsi myocutaneous flap (*c* and *d*). Overall esthetic assessment improved from “poor” to “fair.”

Before reconstruction, we found that all factors related to patient profile (ie, age, body mass index, preoperative brassiere cup size, and tumor location) did not affect the preoperative esthetic scores (*P* > .3, for each factor; data not shown). Table 4 shows the postoperative overall esthetic outcomes by factors related to patient profile. Age, period between BCS and LDM flap, and body mass index did not affect the postoperative esthetic outcome (*P* = .72, .22, and .95, respectively). Patients with large breasts (Fig 1) and those with small breasts (Fig 2) showed comparable postoperative esthetic results (*P* = .21). Tumors located mainly in the lower quadrants of the breast (Fig 3) tended to result in poorer postoperative esthetic outcomes than those in the upper quadrants (*P* = .08). In those patients, scores for both breast shape and scar showed significantly lower values (median value: 1 and 0.8, respectively) than those in patients with tumors in the upper quadrants (median value: 2 and 1.7, respectively) (*P* = .04 and .02, respectively; Mann-Whitney *U* test).

## DISCUSSION

Two major goals of BCS are to achieve local control of the tumor and to preserve the esthetic appearance of the breast. However, it may occasionally be difficult for the surgeon to meet both of these goals. Several factors that could affect esthetic results after BCS have been suggested (ie, tumor size, tumor location and its proximity with the NAC and skin, and breast size and shape).^2^ When these risk factors predict poor postoperative esthetic outcomes, application of immediate oncoplastic surgery (OPS) would decrease such risks.^10^^-^12 Although the concept of OPS has acquired widespread acceptance in the last 2 decades, around 30% of BCS patients are expected to suffer from unfavorable esthetic results.^2^^,^^12^ This is possibly attributed to the relatively difficult planning of OPS in BCS, as well as insufficient collaboration between the breast oncologic surgeon and the plastic surgeon.^13^

In corrective surgery after BCS, the use of a local flap or an implant frequently yields a high rate of postoperative complications because of the nature of irradiated tissue,^14^^,^^15^ resulting in poor esthetic outcomes.^3^ In such situations, the use of a well-vascularized flap such as the LDM flap would lower the rate of postoperative complications,^16^ consequently improving the final esthetic result. In the present study, we reviewed our experience in secondary breast reconstruction with the LDM flap after BCS to assess its validity to improve esthetic outcomes. Importantly, we experienced no major recipient-site– or donor-site–related complication in the early and late course, confirming the safety of using the LDM flap on irradiated breast.^17^ Before reconstruction, although the NAC was preserved in all patients, obvious breast deformities were present because of an extensive contracture, with 13 patients (87%) ranked as poor or fair. In reconstruction, release of scar contracture followed by augmentation with the LDM flap significantly restored breast symmetry, as confirmed by the postoperative result that 13 patients (87%) were ranked as excellent, very good, or good. Interestingly, scores for breast scar also increased in 14 patients in spite of the skin paddle exposure to the surface in 13 patients, which gives a patch-like impression. This indicates that breast deformities could also negatively affect breast scars. Among 7 criteria, scores for NAC size/shape and NAC color did not show significant change before and after reconstruction. It is not hard to expect this result since NAC has been preserved in all patients, and this leads to further credibility to the reliability of our findings.

We found that preoperative brassiere cup size did not affect the overall outcome. In general, small breasts without ptosis tend to result in poor esthetic outcomes after BCS, and these are considered as relative contraindications for immediate OPS without flaps because of insufficient residual tissue.^2^^,^^12^ For those patients, an LDM flap, which can be applied to a small- to medium-sized defect, could be an optimal option in both immediate and delayed reconstruction after BCS (Fig 1).^8^ On the contrary, immediate OPS such as reductive mammoplasty could often be a good option for patients with large breasts with ptosis.^2^ In secondary reconstruction, however, high complication rates due to mobilization of irradiated tissue would make us reluctant to perform them. In our series, patients with larger preoperative breasts were also treated with the LDM flap successfully with no major complications (Fig 2).

In BCS, immediate OPS for tumors located in lower quadrants is relatively challenging, particularly in patients with medium to small breasts. In those patients, insufficiency of residual tissue in the lower pole could make it difficult to create a smooth inframammary fold. In our series, esthetic scores for breast shape and scar were low in those patients. Drooping of the flap often occurs in the late course, which represents double bubble-like deformity (Fig 3). The best way to solve this problem would be to perform immediate OPS with a flap, such as the LDM flap.

## SUMMARY

We reviewed our series of secondary breast reconstruction with the LDM flap after BCS. Analysis of esthetic outcomes by photographic assessment showed that the overall esthetic scores significantly increased postoperatively, regardless of age, period between BCS and LDM flap, body mass index, and breast size. Although exposure of the skin paddle to the skin surface is inevitable in most cases and reconstruction of the lower quadrants is relatively challenging, the LDM flap could be a reliable option to correct breast deformity after BCS.

## Figures and Tables

**Table 1:** Patient data

Patient	Age (BCS), y	Age (LDM), y	Months between	Tumor location	Brassiere cup size	BMI, kg/m^2^	Exposure of skin paddle	Follow-up, mo	Complication
1	53	55	17	Upper	B	19.5	Yes	19	Donor-site seroma
2	46	50	48	Central	C	22.0	Yes	14	None
3	62	64	27	Upper	C	24.9	Yes	105	None
4	50	57	102	Upper	C	22.5	Yes	59	None
5	47	52	65	Upper	A	20.7	Yes	79	None
6	43	44	27	Upper	C	22.1	Yes	65	None
7	38	39	9	Upper	A	19.7	No	82	None
8	56	57	11	Upper	C	Unknown	Yes	62	Donor-site seroma
9	55	59	43	Lower	C	21.3	Yes	15	None
10	38	39	7	Lower	B	20.3	No	121	Donor-site seroma
11	47	50	30	Lower	C	20.1	Yes	16	None
12	45	48	31	Lower	C	23.2	Yes	28	None
13	47	55	89	Upper	C	21.1	Yes	12	None
14	48	55	78	Lower	A	19.1	Yes	141	None
15	41	46	51	Lower	C	22.0	Yes	22	Donor-site seroma

BCS indicates breast-conserving surgery; LDM, latissimus dorsi myocutaneous flap; BMI, body mass index.

**Table 2:** Visual analog scale*

Breast size	2 (symmetric) to 0 (asymmetric)
Breast shape	2 (symmetric) to 0 (asymmetric)
Breast scar	2 (barely visible) to 0 (clearly visible)
NAC size, shape	1 (symmetric) or 0 (asymmetric)
NAC color	1 (symmetric) or 0 (asymmetric)
NAC position	1 (symmetric) or 0 (asymmetric)
Most inferior point of breast	1 (symmetric) or 0 (asymmetric)


NAC indicates nipple-areola complex.

*Overall: 10 to 9 (excellent), 8 to 7 (very good), 6 to 5 (good), 4 to 3 (fair), 2 to 0 (poor).

**Table 3:** Changes in pre- and postoperative esthetic scores*

Criteria	Preoperative	Postoperative	*P*
Breast size	0.33	2.0	**<.0001**
Breast shape	0	1.3	**.0001**
Breast scar	0.50	1.0	**.008**
NAC size, shape	0.33	0.67	.13
NAC color	1.0	1.0	.17
NAC position	0.33	1.0	**.0013**
Most inferior point of breast	0	0.67	**.0005**
Overall	2.0	7.5	**<.0001**

NAC indicates nipple-areola complex.

*Median values are presented for each criterion. Mann-Whitney *U* test for preoperative values versus postoperative values.

**Table 4:** Factors affecting esthetic outcome*

	Excellent	Very good	Good	Fair	Poor	*P*
Age, y						
<50	2 (33%)	2 (33%)	1 (17%)	1 (17%)	0	.72†
≥50	2 (22%)	3 (33%)	3 (33%)	1 (11%)	0	
Months between BCS and LDM						
<30	3 (50%)	1 (17%)	2 (33%)	0	0	.22†
≥30	1 (11%)	4 (44%)	2 (22%)	2 (22%)	0	
BMI, kg/m^2^						
<22	2 (22%)	3 (33%)	3 (33%)	1 (11%)	0	.95†
≥22	1 (25%)	2 (50%)	1 (25%)	1 (25%)	0	
Preoperative brassiere cup size						
A	1 (33%)	0	1 (33%)	1 (33%)	0	.21‡
B	0	0	2 (100%)	0	0	
C	3 (30%)	4 (40%)	2 (20%)	1 (10%)	0	
Tumor location						
Upper quadrants	3 (33%)	4 (44%)	2 (22%)	0	0	.08†
Lower quadrants	1 (17%)	1 (17%)	2 (33%)	2 (33%)	0	

BCS indicates breast-conserving surgery; BMI, body mass index; LDM, latissimus dorsi myocutaneous flap.

*Values in parentheses indicate the percentage of patients in a specific group.

†Mann-Whitney *U* test for age, months between BCS and LDM, BMI, tumor location versus esthetic outcome.

‡Spearman rank correlation coefficient for preoperative brassiere cup size versus esthetic outcome.
